# Time-Dependent ROC Curve Analysis for Assessing the Capability of Radiation-Induced CD8 T-Lymphocyte Apoptosis to Predict Late Toxicities after Adjuvant Radiotherapy of Breast Cancer Patients

**DOI:** 10.3390/cancers15194676

**Published:** 2023-09-22

**Authors:** Célia Touraine, Audrey Winter, Florence Castan, David Azria, Sophie Gourgou

**Affiliations:** 1 Biometrics Unit, Cancer Institute of Montpellier (ICM), University Montpellier, 34090 Montpellier, France; celia.touraine@icm.unicancer.fr (C.T.); florence.castan@icm.unicancer.fr (F.C.); sophie.gourgou@icm.unicancer.fr (S.G.); 2 French National Platform Quality of Life and Cancer, 34090 Montpellier, France; 3 Desbrest Institute of Epidemiology and Public Health (IDESP), University Montpellier, INSERM, 34090 Montpellier, France; 4 Radiotherapy Unit, Cancer Institute of Montpellier (ICM), University Montpellier, 34090 Montpellier, France; david.azria@icm.unicancer.fr

**Keywords:** ROC curve, time-dependent AUC, biomarker, prediction, radiotherapy, breast fibrosis

## Abstract

**Simple Summary:**

Intrinsic radiosensitivity has been found to increase the risk of radiation-induced toxicities. Identifying individual characteristics that can predict the risk of late fibrosis in breast cancer patients is essential to better adapt the irradiation dose to be delivered. Previous studies found radiation-induced CD8 T-lymphocyte apoptosis (RILA) to be associated with grade ≥2 late toxicities, as well as tobacco smoking status and adjuvant hormonotherapy. In this article we evaluate the predictive performance of the RILA, alone and in association with the other factors, using a recent ROC curve approach suitable to the dynamic nature of fibrosis occurrence. Our analysis confirmed the RILA predictive ability, which was not necessarily improved by the others factors. This article also illustrates the underused time-dependent ROC curve method.

**Abstract:**

Late fibrosis can occur in breast cancer patients treated with curative-intent radiotherapy. Predicting this toxicity is of clinical interest in order to adapt the irradiation dose delivered. Radiation-induced CD8 T-lymphocyte apoptosis (RILA) had been proven to be associated with less grade ≥2 late radiation-induced toxicities in patients with miscellaneous cancers. Tobacco smoking status and adjuvant hormonotherapy were also identified as potential factors related to late-breast-fibrosis-free survival. This article evaluates the predictive performance of the RILA using a ROC curve analysis that takes into account the dynamic nature of fibrosis occurrence. This time-dependent ROC curve approach is also applied to evaluate the ability of the RILA combined with the other previously identified factors. Our analysis includes a Monte Carlo cross-validation procedure and the calculation of an expected cost of misclassification, which provides more importance to patients who have no risk of late fibrosis in order to be able to treat them with the maximal irradiation dose. Performance evaluation was assessed at 12, 24, 36 and 50 months. At 36 months, our results were comparable to those obtained in a previous study, thus underlying the predictive power of the RILA. Based on specificity and cost, RILA alone seemed to be the most performant, while its association with the other factors had better negative predictive value results.

## 1. Introduction

Given the long survival of early stage breast cancer patients receiving adjuvant radiotherapy (RT) after breast-conserving surgery, radiation oncologists are especially concerned with prediction and prevention of late toxicities [1,2,3]. These include late breast fibrosis, which manifests itself through several symptoms such as muscle shortening, atrophy, pain, and skin induration and thickening [4,5]. This side effect is considered irreversible with a strong negative impact on health-related quality of life, as it may cause long-term disability and a lot of discomfort. While the radiation dose is prescribed according to clinical scenarios in current practice, identifying individual characteristics that can predict the risk of late fibrosis is essential to better adapt the dose to be delivered. In particular, intrinsic radiosensitivity has been found to increase the risk of radiation-induced toxicities [6]. A rapid radiosensitivity assay, based on the flow cytometric assessment of radiation-induced CD8 T-lymphocyte apoptosis (RILA), has been developed [7,8,9], and a prospective study found that a high RILA value was associated with less grade ≥2 late radiation-induced toxicities [10] in patients with miscellaneous cancers. The ability of the RILA to predict grade ≥2 late toxicities was confirmed in two prospective multicenter trials (NCT00893035) in prostate and breast cancer.

In the latter, RILA was found significantly lower (p = 0.004) in patients with late breast fibrosis (med = 10.9, range 1.3–35.9) at 3 years than in patients without late breast fibrosis (med = 15.6, range 0.7–52.8) [11] at 3 years. A receiver operating characteristics (ROC) curve analysis [12,13,14] found an area under the ROC curve (AUC) of 0.62 (95% CI 0.54–0.70). In univariate analyses, a shorter late-breast-fibrosis-free survival was found for decreasing values of RILA (p = 0.005, variable treated as continuous), and in patients with RILA < 12 as compared to patients with RILA ≥ 12 (p = 0.001, variable treated as categorical). In the multivariate regression analysis, tobacco smoking and adjuvant hormonotherapy (HT) were retained additionally to RILA as factors associated with late-breast-fibrosis-free survival. 

However, the ability of the factors identified in the multivariate analysis to help RILA to predict late fibrosis was not studied. Additionally, the ROC curve analysis of the RILA alone was succinct and did not take into account the dynamic nature of fibrosis occurrence. The aim of the present work was to apply a time-dependent ROC curve approach [15,16] in order to better evaluate the ability of the RILA to predict the risk of late fibrosis, in association or not with other individual characteristics.

## 2. Materials and Methods

### 2.1. The Clinical Trial Data

We analyzed data from the study registered with ClinicalTrials.gov (number NCT00893035), which consisted of two prospective multicenter trials (breast cancer and prostate cancer) to evaluate RILA as a predictor of late toxicity after RT. This article focuses on the breast cancer cohort. A total of 502 breast cancer patients treated by conservative surgery and adjuvant RT were recruited in ten French centers from January 2007 to July 2011. HT and/or chemotherapy were possibly part of the adjuvant treatment. Inclusion/exclusion criteria included T1/T2 tumors, no metastases, and negative surgical margins. After inclusion (after surgery and before RT), a blood sample was collected from each patient and RILA was assessed by flow cytometry. Toxicities were assessed and graded according to the classification CTCAE v3.0 [17]: before RT, every week during RT (8 weeks of RT), 1, 3, and 6 months after the last RT fraction (M1, M3, M6), and every 6 months up to 3 years after the last RT fraction (M12, M18, M24, M30, M36). The primary endpoint was defined as the most severe late breast fibrosis of grade ≥2 observed from 3 months to 3 years after RT. We considered the first day of RT as baseline time, up until late breast fibrosis was defined by the delay between first observation of a late breast fibrosis and the start of RT. A total of 456 patients (treated and with blood sample available) were included in the final analysis; among which 434 patients reached the 3 years post-RT assessment; the planned follow-up of the other 22 patients was interrupted due to consent withdrawal (*n* = 3), lost to follow-up (*n* = 7), death (*n* = 4), tumor recurrence (*n* = 5), mastectomy (*n* = 2), or second cancer (*n* = 1).

### 2.2. The Time-Dependent ROC Curve Approach

Studying the ability of a marker to predict the occurrence of a time-dependent event is generally performed using a standard ROC curve analysis. In practice, a time frame is fixed, let us say [$t_{0}$,$\;t_{max}$], where $t_{0}$ is usually the time of inclusion ($t_{0}=0$) and $t_{max}$ the study duration. The population is divided between those who had experienced the event within [$t_{0}$,$\;t_{max}$] and those who did not. The standard ROC curve approach can then be applied to assess how performant the marker is to discriminate the population at time point $t_{max}$. However, such an analysis can only include patients for whom the event status is known at that time; that is, right-censored patients with a time-to-event before $t_{max}$ must be excluded from the analysis. 

Recent research has developed a time-dependent ROC curve approach that has been shown to be more effective than the standard approach in this context [15]. The event occurrence is not considered as fixed anymore: it is not only the event status at $t_{max}$ that is taken into account but also the time between $t_{0}$ and $t_{max}$, i.e., the delay at which the event occurred (if so). As a consequence, individuals that experienced the event before $t_{max}$ are not treated in the same way depending on whether they have experienced it at an early or later time. In fact, the predicted variable is not a binary variable anymore but a survival variable. For the individuals who did not experience the event before $t_{max}$, the time-to-event is right-censored at the time of last observation so that they are allowed to experience it later. Note that this last point allows taking into account the individuals lost to follow-up before $t_{max}$, whereas they would be excluded from the analysis using the standard approach.

### 2.3. Statistical Analysis

#### 2.3.1. Prediction

Late breast fibrosis was defined as grade ≥2 observed at least 3 months post-RT. Let us denote by bf+ and bf−, respectively, for patients with and without late breast fibrosis at time t. The goal was to discriminate between bf+ and bf− patients at a given time t using either the baseline RILA alone or a set of individual baseline characteristics including RILA.

Using the RILA alone to predict late fibrosis consisted in determining a threshold to distinguish between bf+ and bf− patients. To include other individual characteristics in the prediction, the first step was to model the relationship between the time-dependent fibrosis status and the potential predictors. To achieve this goal, a survival regression model was used instead of a logistic regression model, as performed in the standard ROC curve approach. We considered a regression Cox model [18] for the time to late breast fibrosis

(1) $$h_{i}\left(t\right)=h_{0}\left(t\right)lp_{i}$$

where $h_{0}$ was the baseline hazard function and $lp_{i}$ was the linear predictor of patient i. Following the final model retained in the previous publication [11], we used the linear predictor

(2) $$lp_{i}=exp\left(\beta_{1}Z_{i1}+\beta_{2}Z_{i2}+\beta_{3}Z_{i3}\right)$$

where $Z_{i1}$ was the baseline RILA value of patient i, $Z_{i2}$ its tobacco smoking status (active/former vs. no) and $Z_{i3}$ was its adjuvant HT status (yes vs. no). The linear predictor estimate was provided by ${\hat{lp}}_{i}={exp(\hat{\beta }}_{1}Z_{i1}+{\hat{\beta }}_{2}Z_{i2}+{\hat{\beta }}_{3}Z_{i3})$ with ${\hat{\beta }}_{1}$, ${\hat{\beta }}_{2}$ and ${\hat{\beta }}_{3}$ as the estimates of the Cox model coefficients. Then, just as with the RILA alone, a threshold below/above which patient i is predicted as bf+/bf− needed to be determined.

Hereafter, we will denote by $M_{i}$ the marker for patient i, which can refer to the RILA value alone or the score derived from the Cox model ($lp_{i}$).

#### 2.3.2. Endpoints

For a given threshold c, the time-dependent sensitivity (*Se*) and specificity (*Sp*) were defined by, respectively

$$Se\left(c,t\right)=P\left(M_{i}>c|T_{i}\leq t\right)$$

$$Sp\left(c,t\right)=P\left(M_{i}\leq c|T_{i}>t\right)$$

where $T_{i}$ represented the time to late fibrosis. Therefore, sensitivity at time t corresponded to the probability of a patient having a marker value above the threshold c, given that this patient had late breast fibrosis before t (true positive). Specificity at time t corresponded to the probability of a patient having a marker value below (or equal to) the threshold, given that this patient was still without late breast fibrosis at time t (true negative). To estimate *Se* and *Sp*, the nearest neighbor estimator of Heagerty et al. [6] was used, which corrects some drawbacks of the Kaplan–Meier estimator of Heagerty et al. [16]. Indeed, the latter does not guarantee Se and Sp to be monotone and bounded in [0, 1], and does not apply to situations of marker-dependent censoring.

Estimating *Se* and *Sp* served us in order to plot the time-dependent ROC curve at a specific time t, that is, the graphical display of $\hat{Se}\left(c,t\right)$ against one minus $\hat{Sp}\left(c,t\right)$ for all possible threshold values c [19,20,21,22].

To evaluate the performance of the marker, we calculated the time-dependent AUC, which corresponds to the probability that a bf+ patient has a higher marker value before t than a patient still bf− at t; its value varies from 0.5 (not informative) to 1 (perfect discrimination) [23,24,25]. The most discriminating threshold was determined by maximizing the Youden’s index: $Se\left(c,t\right)$ + $Sp\left(c,t\right)$ − 1 [26].

Once the threshold was determined, we calculated other performance metrics, including positive predictive value (PPV) and negative predictive value (NPV), which estimate the probability that a patient will/will not have fibrosis given that she has been predicted as bf+/bf− [27]. They can be easily calculated using the number of true negative (TN), the number of true positive (TP), the number of false negative (FN), and the number of false positive (FP): NPV = TN/(TN + FN) and PPV = TP/(TP + FP). Contrary to sensitivity and specificity where the fibrosis status of the patient is known, they allow us to assess the probability of being right (or wrong) in practice when predicting the late fibrosis status bf+/bf− of a patient from its RILA value. However, they have the disadvantage of being dependent of the prevalence.

**Figure 1:**
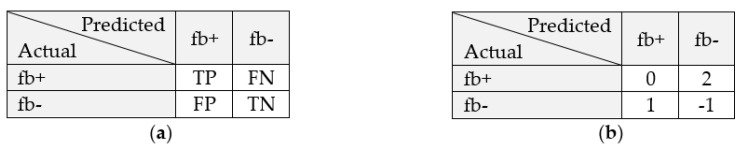
Confusion matrix (**a**) and cost matrix (**b**).

Finally, we calculated an expected cost of misclassification. We chose the cost matrix depicted in Figure 1b in relation to the correspondent confusion matrix (Figure 1a).

In order to meet the aforementioned objectives and to be as close as possible to clinicians needs, weights were chosen in the cost matrix. Indeed, incorrect classifications did not have the same consequences; by giving more weight to *FN* than *FP*, we consider that by predicting that a patient will not have late breast fibrosis while she is at high risk of developing it (i.e., *FN*) is more damageable than predicting that a patient will have a fibrosis while she is at a low risk (i.e., *FP*). Similarly, correct classifications were not considered the same, as it was more important to detect patients who have no risk of developing late breast fibrosis (i.e., *TN*) in order to treat them with the maximal irradiation dose than to detect patients who will develop late breast fibrosis (i.e., *TP*). In the cost matrix, we thus favored well-classified fb- rather than well-classified fb+ (i.e., −1 weight for *TN* and 0 for *TP*). 

The expected cost of misclassification C was obtained as a weighted mean of the components of the confusion matrix using the components of the cost matrix as weights, with the best-performing model being the one that minimizes C:

$$C=\frac{1}{N}\left(TP\times 0+FN\times 2+FP\times 1+TN\times \left(-1\right)\right)$$

### 2.4. Methodology of Analysis

We first used the same population to both train and evaluate the model in order to obtain results comparable to those of the previous ROC curve analysis (i.e., the standard approach), which had been performed in all of the population [11]. In a second step, we split the data into training and validation sets in a cross validation procedure. We performed three methods of cross validation: K-fold with K = 5 subsamples, leave-one-out and Monte-Carlo [28,29]. They all provided similar results and, here, we present the results of the latter. Specifically, the following procedure was repeated 500 times; (1) randomly splitting the data into a training set (2/3 of the population) and a validation set (the remaining 1/3); (2) fitting the model to the training data and estimating the optimal threshold; (3) assessing predictive accuracy on the validation data using the estimations from the model fitted on the training data. The results were then averaged over the 500 splits. Of note, resampling techniques allow for evaluating model performances and are recommended for prediction model development.

### 2.5. Software

All analyses were performed using R software version 4.3.0 [30]. The survival package was used to fit the Cox model [31] and the survivalROC package to estimate time-dependent ROC curves and model performances [32].

## 3. Results

Among the 456 patients, 61 late breast fibrosis occurred. Time to late breast fibrosis was right-censored at the last assessment time without late breast fibrosis; 22 patients had a right-censored time prior to their last planned assessment visit of 3 years post-RT (M36). We performed the aforementioned analysis at 12, 24 and 36 months. At these respective times, late breast fibrosis had occurred in 35, 49, 55 patients, and the Kaplan–Meier estimates of fibrosis-free survival rate were of 0.923 (95% confidence interval (CI) [0.899, 0.948]), 0.892 (95% CI [0.863, 0.921]), 0.878 (95% CI [0.848, 0.909]). We also performed an analysis at 50 months, i.e., when all the 61 breast fibrosis events had been observed; the Kaplan–Meier estimate of fibrosis-free survival rate was of 0.836 (95% CI [0.783, 0.893]). Indeed, though the last evaluation was planned 36 months after the end of RT. In practice, the evaluations varied from patient to patient in such a way that the last fibrosis event was observed at 48.8 months.

### 3.1. Baseline RILA Alone

**Figure 2:**
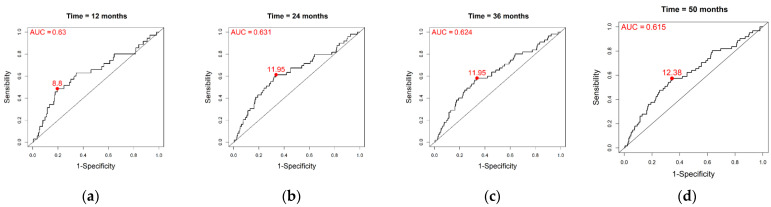
Time-dependent ROC curves for the RILA marker along with AUC and optimal threshold according to the Youden’s index obtained from the overall population (*n* = 456) at: (**a**) 12 months; (**b**) 24 months; (**c**) 36 months; (**d**) 50 months.

Figure 2 depicts the time-dependent ROC curves obtained when using the baseline RILA alone as a marker (the used variable being RILA multiplied by −1 in order to associate low values of RILA with an increased risk of late breast fibrosis), and Table 1 shows its performances over time alongside their means across the 500 Monte Carlo splits, which are concordant with the one obtained in the overall population. The AUC at 12, 24, 36 and 50 months were, respectively, 0.630, 0.631, 0.624 and 0.615. Of note, using the Kaplan–Meier estimator of Heagerty, they were 0.631, 0.632, 0.625 and 0.527, suggesting RILA-dependent censoring after 36 months. The optimal RILA thresholds according to the Youden’s index were, respectively, 8.8, 11.95, 11.95 and 12.38; that is, a threshold of 8.8 would better discriminate between bf+ and bf− patients in the year post-RT, while from the second year post-RT, a threshold around 12 seems to better discriminate between whether or not a patient will experience late breast fibrosis.

Of note, there were 35, 49, 55 and 61 events and 4, 9, 34, 367 censorships out of the 456 patients at 12, 24, 36 and 50 months, respectively.

### 3.2. Composite Marker

The coefficient estimates of the multivariate Cox model presented in Section 2.3.1 are shown in Table 2. 

Baseline RILA and adjuvant HT were found to be significantly associated with the risk of late breast fibrosis; for example, having adjuvant HT led to a 3.17-fold increase in the risk of late breast fibrosis. 

**Figure 3:**
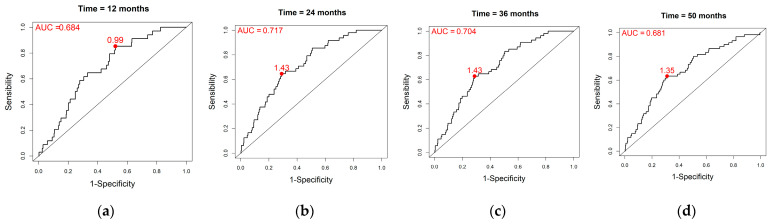
Time-dependent ROC curves for the composite marker (combining baseline RILA, tobacco smoking status and adjuvant HT status) along with AUC and optimal threshold according to the Youden’s index obtained in the overall population (*n* = 456) at: (**a**) 12 months; (**b**) 24 months; (**c**) 36 months; (**d**) 50 months.

Using the estimates from Table 2, the linear predictor was calculated for each patient. As for baseline RILA alone, time-dependent ROC curves were drawn using the composite marker lp, combining baseline RILA, tobacco smoking status and adjuvant HT status (see Figure 3). The AUC at 12, 24, 36 and 50 months were, respectively, 0.684, 0.717, 0.704 and 0.681, with optimal lp thresholds of 0.99, 1.43, 1.43 and 1.35. The performances at each time point can be found in Table 3 as well as their means using the Monte Carlo method. Among the 436 patients (i.e., without missing values for tobacco smoking status and adjuvant HT status), there were 34, 48, 54 and 60 events, and 4, 9, 33 and 348 censorships at month 12, 24, 36 and 50, respectively. 

### 3.3. Baseline RILA Alone and Composite Marker Comparison

The baseline RILA alone and the composite marker had quite similar performances across time, namely a higher specificity than sensitivity (i.e., they better identified patients without toxicity), except at month 12 where the composite marker was able to better identify bf+ patients (i.e., higher sensitivity). This observation was confirmed when it came to the Monte Carlo simulations. The prevalence being low (i.e., 61 bf+/456), as expected, meant low PPV and high NPV were observed. Based on the Monte Carlo simulations, RILA alone had better performances in regard to specificity and cost, while the multivariate Cox model was better in terms of Se, AUC, PPV and NPV results. 

## 4. Discussion

RILA is a radiation-induced lymphocytic apoptosis, which is responsible of the rapid disappearance of lymphocytes after total, therapeutic or accidental body irradiation. This rate of apoptosis is very heterogeneous among the population; in particular, it was observed at a spontaneous rate three times higher than normal in AT heterozygous patients (i.e., potentially hyper-radiosensitive) [33]. It has been shown in several studies [5,6,11,34,35,36,37,38,39] that low RILA is associated with late toxicities in various types of cancer. Of note, even if a relationship has been highlighted between RILA and late toxicities, the underlying mechanisms of this association are still unclear [5]. Other factors influencing late toxicities were also studied [40,41,42,43,44], among them smoking status and adjuvant HT. In this work, we evaluated the ability of RILA (alone and combined with smoking status and adjuvant HT) to predict late fibrosis in breast cancer patients treated with curative-intent RT using the recent time-dependent ROC curve approach. We also assessed their performances through a cross-validation procedure. 

A succinct ROC curve analysis had been previously performed in the same population to evaluate the ability of the RILA to predict late fibrosis at 3 years [11]. It led to an AUC of 0.620 (95% CI [0.54; 0.70]) and with a cut-off of 12% (arbitrarily chosen as the first tercile), a Se of 56%, a Sp of 67%, a PPV of 22% and an NPV of 90%. Our results at 36 months were similar to the previous results, with an AUC of 0.624, a Se of 58%, a Sp of 66%, a PPV of 19% and an NPV of 92% (the RILA cutoff being 11.95% based on Youden’s index). The same results were obtained when rounding the cutoff to 12%, thus justifying the choice made in the previous study. Of note, we found that a cutoff of 8.8 made it possible to predict early events (late fibrosis within the first year after RT). Moreover, in this article, we proposed an ROC curve analysis based on a predictive score corresponding to a composite marker derived from a regression incorporating the RILA and other individual characteristics. Note that the previous ROC analysis implied fixing a maximal time of observation of the fibrosis status (M36 visit) and classifying the patients between those who experienced fibrosis between 3 months and M36 visit and those who were followed up until this visit without fibrosis at this date. Thus, it included the 435 evaluable patients who were followed up to M36 (*n* = 434) or who presented a late toxicity before being lost to follow-up (*n* = 1), while our time-dependent approach included all of the population for analysis (*n* = 456), whatever the time point considered for the evaluation. Of note, in our analysis, the timeline considered was different from the one in the original article [11] in which they used “discrete” times (i.e., the visit), whereas we considered continuous time (made possible by the time-dependent approach). Contrary to the previous publication, we used a cross-validation procedure to confirm the results obtained in all the population. Nevertheless, in the future, it could be interesting to perform an external validation [45].

In general, the use of time-dependent ROC curve shows several advantages. As previously noted, it allows the inclusion of all patients in the analysis (i.e., lost to follow-up patients do not have to be excluded), and predictions can be easily performed as well as model performances estimated at different time points, the status of the event of interest (late fibrosis status in this article) being automatically updated. Note that, even if this fact was not exploited in our application, an important advantage of the time-dependent ROC curve approach is to enable being able to take into account time-varying markers and evaluating their predictive performances over time [46]. Thus, to make a prediction at a given time point t, based on a certain marker that has been longitudinally assessed, it becomes possible to use all the marker trajectory until time t instead of only its baseline value.

## 5. Conclusions

In this article, we performed an in-depth analysis of the RILA performance to predict late fibrosis in breast cancer patients, in association or not with other individual characteristics, using a time-dependent ROC curve approach. We found that the optimal RILA threshold to assist clinicians in the therapeutic decision in adapting the irradiation dose was 12. As the main objective was to detect patients who will not have late fibrosis, specificity, NPV and cost were the most important criteria to be taken into account. Based on the Monte Carlo simulations, RILA alone seemed to be the most performant in terms of specificity and cost, while the composite marker combining RILA, smoking status and adjuvant HT status had better NPV results. Of note, if the matter lies on the likelihood that the test can differentiate between fb+ and fb− patients (i.e., the accuracy of negative results) one should get more interest in NPV while interest should be focused on specificity if the adequacy of the screening test is of interest [47].

The standard ROC curve approach is often used to assess the ability of a baseline marker to predict a certain event occurring over time. This article illustrates the underused time-dependent ROC curve approach, which takes into account the dynamic nature of the event occurrence and allows the consideration of time-varying markers. 

## Figures and Tables

**Table 1:** RILA alone performances estimated in the overall population (*n* = 456) and using Monte Carlo simulations at 12, 24, 36 and 50 months (i.e., mean performances).

	Time	Threshold	AUC	Se	Sp	PPV	NPV	Cost
Overall population	*t* = 12	8.8	0.630	0.486	0.805	0.172	0.950	−0.485
	*t* = 24	11.95	0.631	0.612	0.663	0.180	0.934	−0.208
	*t* = 36	11.95	0.624	0.582	0.663	0.192	0.920	−0.186
	*t* = 50	12.38	0.615	0.574	0.656	0.205	0.909	−0.156
Monte Carlo simulations	*t* = 12	10.08	0.630	0.478	0.734	0.134	0.945	−0.353
	*t* = 24	11.08	0.632	0.515	0.695	0.168	0.924	−0.243
	*t* = 36	10.91	0.624	0.478	0.702	0.181	0.908	−0.230
	*t* = 50	11.51	0.617	0.482	0.678	0.191	0.895	−0.170

**Table 2:** Multivariate Cox model (*n* = 436, 60 bf+).

Variable	Coefficient	HR [95% CI]	*p*-Value
Baseline RILA *	0.04	1.04 [1.01–1.08]	0.012
Tobacco smoking status (active/former vs. no)	0.44	1.56 [0.93–2.60]	0.091
Adjuvant HT status (yes vs. no)	1.15	3.17 [1.36–7.40]	0.008

* multiplied by −1 in order to associate low values of RILA with an increased risk of late breast fibrosis.

**Table 3:** Composite marker performances estimated in the overall population (*n* = 436) and using Monte Carlo simulations at 12, 24, 36 and 50 months (i.e., mean performances).

	Time	Threshold	AUC	Se	Sp	PPV	NPV	Cost
Overall population	*t* = 12	0.99	0.684	0.853	0.480	0.261	0.975	0.060
	*t* = 24	1.43	0.717	0.646	0.709	0.215	0.942	−0.294
	*t* = 36	1.43	0.704	0.630	0.712	0.236	0.932	−0.280
	*t* = 50	1.35	0.681	0.633	0.689	0.245	0.922	−0.225
Monte Carlo simulations	*t* = 12	1.33	0.664	0.614	0.616	0.121	0.951	−0.153
	*t* = 24	1.39	0.694	0.626	0.660	0.190	0.936	−0.203
	*t* = 36	1.34	0.683	0.627	0.642	0.201	0.926	−0.156
	*t* = 50	1.46	0.663	0.600	0.662	0.223	0.913	−0.169

## Data Availability

The dataset is not publicly available due to confidentiality requirements. The R scripts to perform the data analysis are available from the authors upon request.

## References

[1] Darby S.C., Ewertz M., McGale P., Bennet A.M., Blom-Goldman U., Brønnum D., Correa C., Cutter D., Gagliardi G., Gigante B. (2013). Risk of ischemic heart disease in women after radiotherapy for breast cancer. N. Engl. J. Med..

[2] Ferini G., Molino L., Tripoli A., Valenti V., Illari S.I., Marchese V.A., Cravagno I.R., Borzi G.R. (2021). Anatomical Predictors of Dosimetric Advantages for Deep-inspiration-breath-hold 3D-conformal Radiotherapy Among Women with Left Breast Cancer. Anticancer. Res..

[3] Ferini G., Valenti V., Viola A., Umana G.E., Martorana E. (2022). A Critical Overview of Predictors of Heart Sparing by Deep-Inspiration-Breath-Hold Irradiation in Left-Sided Breast Cancer Patients. Cancers.

[4] Straub J.M., New J., Hamilton C.D., Lominska C., Shnayder Y., Thomas S.M. (2015). Radiation-induced fibrosis: Mechanisms and implications for therapy. J. Cancer Res. Clin. Oncol..

[5] Fhoghlú M.N., Barrett S. (2019). A Review of Radiation-Induced Lymphocyte Apoptosis as a Predictor of Late Toxicity after Breast Radiotherapy. J. Med. Imaging Radiat. Sci..

[6] Azria D., Betz M., Bourgier C., Sozzi W.J., Ozsahin M. (2012). Identifying patients at risk for late radiation-induced toxicity. Crit. Rev. Oncol..

[7] Bordón E., Henríquez-Hernández L.A., Lara P.C., Ruíz A., Pinar B., Rodríguez-Gallego C., Lloret M. (2010). Prediction of clinical toxicity in locally advanced head and neck cancer patients by radio-induced apoptosis in peripheral blood lymphocytes (PBLs). Radiat. Oncol..

[8] Foro P., Algara M., Lozano J., Rodriguez N., Sanz X., Torres E., Carles J., Reig A., Membrive I., Quera J. (2014). Relationship between radiation-induced apoptosis of T lymphocytes and chronic toxicity in patients with prostate cancer treated by radiation therapy: A prospective study. Int. J. Radiat. Oncol..

[9] Schnarr K., Boreham D., Sathya J., Julian J., Dayes I.S. (2009). Radiation-induced lymphocyte apoptosis to predict radiation therapy late toxicity in prostate cancer patients. Int. J. Radiat. Oncol..

[10] Ozsahin M., Crompton N.E., Gourgou S., Kramar A., Li L., Shi Y., Sozzi W.J., Zouhair A., Mirimanoff R.O., Azria D. (2005). CD4 and CD8 T-Lymphocyte Apoptosis Can Predict Radiation-Induced Late Toxicity: A Prospective Study in 399 Patients. Clin. Cancer Res..

[11] Azria D., Riou O., Castan F., Nguyen T.D., Peignaux K., Lemanski C., Lagrange J.-L., Kirova Y., Lartigau E., Belkacemi Y. (2015). Radiation-induced CD8 T-lymphocyte Apoptosis as a Predictor of Breast Fibrosis After Radiotherapy: Results of the Prospective Multicenter French Trial. EBioMedicine.

[12] Fawcett T. (2006). An introduction to ROC analysis. Pattern Recogn. Lett..

[13] Swets J.A. (1988). Measuring the accuracy of diagnostic systems. Science.

[14] Zou K.H., O’Malley A.J., Mauri L. (2007). Receiver-Operating Characteristic Analysis for Evaluating Diagnostic Tests and Predictive Models. Circulation.

[15] Kamarudin A.N., Cox T., Kolamunnage-Dona R. (2017). Time-dependent ROC curve analysis in medical research: Current methods and applications. BMC Med. Res. Methodol..

[16] Heagerty P.J., Lumley T., Pepe M.S. (2000). Time-Dependent ROC Curves for Censored Survival Data and a Diagnostic Marker. Biometrics.

[17] Trotti A., Colevas A.D., Setser A., Rusch V., Jaques D., Budach V., Langer C., Murphy B., Cumberlin R., Coleman C.N. (2003). CTCAE v3.0: Development of a comprehensive grading system for the adverse effects of cancer treatment. Semin. Radiat. Oncol..

[18] Cox D.R. (1972). Regression Models and Life-Tables. J. R. Stat. Soc. Ser. B Stat. Methodol..

[19] Hanley J.A. (1989). Receiver operating characteristic (ROC) methodology: The state of the art. Crit. Rev. Comput. Tomogr..

[20] Begg C.B. (1991). Advances in statistical methodology for diagnostic medicine in the 1980’s. Stat. Med..

[21] Pepe M., Leisenring W., Rutter C. (2000). Evaluating diagnostic tests in public health. Handbook of Statistics.

[22] Zweig M.H., Campbell G. (1993). Receiver-operating characteristic (ROC) plots: A fundamental evaluation tool in clinical medicine. Clin. Chem..

[23] Pepe M.S. (2004). The Statistical Evaluation of Medical Tests for Classification and Prediction.

[24] Bamber D. (1975). The area above the ordinal dominance graph and the area below the receiver operating characteristic graph. J. Math. Psychol..

[25] Hanley J.A., McNeil B.J. (1982). The meaning and use of the area under a receiver operating characteristic (ROC) curve. Radiology.

[26] Youden W.J. (1950). Index for rating diagnostic tests. Cancer.

[27] Altman D.G., Bland J.M. (1994). Statistics Notes: Diagnostic tests 2: Predictive values. BMJ.

[28] Refaeilzadeh P., Tang L., Liu H., Liu L., Özsu M.T. (2009). Cross-Validation. Encyclopedia of Database Systems.

[29] James G., Witten D.M., Hastie T., Tibshirani R. (2021). An Introduction to Statistical Learning.

[30] R Core Team (2017). R: A Language and Environment for Statistical Computing.

[31] Therneau T.M. (2023). A Package for Survival Analysis in R, R Package Version 3.5-5. https://CRAN.R-project.org/package=survival.

[32] Patrick J. (2022). Heagerty and Packaging by Paramita Saha-Chaudhuri, survivalROC: Time-Dependent ROC Curve Estimation from Censored Survival Data, R Package Version 1.0.3.1. https://CRAN.R-project.org/package=survivalROC.

[33] Duchaud E., Ridet A., Delic Y., Cundari E., Moustacchi E., Rosselli F. (1994). Changes in the radiation-induced apoptotic response in homozygotes and heterozygotes for the ataxia-telangiectasia gene. C. R. Acad. Sci. III.

[34] Mirjolet C., Merlin J., Truc G., Noël G., Thariat J., Domont J., Sargos P., Renard-Oldrini S., Ray-Coquard I., Liem X. (2019). RILA blood biomarker as a predictor of radiation-induced sarcoma in a matched cohort study. EBioMedicine.

[35] Bourgier C., Kerns S., Gourgou S., Lemanski C., Gutowski M., Fenoglietto P., Romieu G., Crompton N., Lacombe J., Pèlegrin A. (2016). Concurrent or sequential letrozole with adjuvant breast radiotherapy: Final results of the CO-HO-RT phase II randomized trial. Ann. Oncol..

[36] Henríquez-Hernández L.A., Carmona-Vigo R., Pinar B., Bordón E., Lloret M., Núñez M.I., Rodríguez-Gallego C., Lara P.C. (2011). Combined low initial DNA damage and high radiation-induced apoptosis confers clinical resistance to long-term toxicity in breast cancer patients treated with high-dose radiotherapy. Radiat. Oncol..

[37] Azria D., Ozsahin M., Kramar A., Peters S., Atencio D.P., Crompton N.E., Mornex F., Pèlegrin A., Dubois J.-B., Mirimanoff R.-O. (2008). Single nucleotide polymorphisms, apoptosis, and the development of severe late adverse effects after radiotherapy. Clin. Cancer Res..

[38] Crompton N.E., Shi Y.-Q., Emery G.C., Wisser L., Blattmann H., Maier A., Li L., Schindler D., Ozsahin H., Ozsahin M. (2001). Sources of variation in patient response to radiation treatment. Int. J. Radiat. Oncol..

[39] Fuentes-Raspall M.J., Caragol I., Alonso C., Cajal T.R.Y., Fisas D., Seoane A., Carvajal N., Bonache S., Díez O., Gutiérrez-Enríquez S. (2015). Apoptosis for prediction of radiotherapy late toxicity: Lymphocyte subset sensitivity and potential effect of TP53 Arg72Pro polymorphism. Apoptosis Int. J. Program. Cell Death. Apoptosis.

[40] Shaitelman S.F., Howell R.M., Smith B.D. (2017). The Effects of Smoking on Late toxicity from breast radiation. J. Clin. Oncol..

[41] Bourgier C., Castan F., Riou O., Nguyen T.-D., Peignaux K., Lemanski C., Lagrange J.-L., Kirova Y., Lartigau E., Belkacemi Y. (2018). Impact of adjuvant hormonotherapy on radiation-induced breast fibrosis according to the individual radiosensitivity: Results of a multicenter prospective French trial. Oncotarget.

[42] Pasquier D., Bataille B., Le Tinier F., Bennadji R., Langin H., Escande A., Tresch E., Darloy F., Carlier D., Crop F. (2021). Lartigau, Correlation between toxicity and dosimetric parameters for adjuvant intensity modulated radiation therapy of breast cancer: A prospective study. Sci. Rep..

[43] Mayer E.L. (2013). Early and Late Long-Term Effects of Adjuvant Chemotherapy. Am. Soc. Clin. Oncol. Educ. Book.

[44] De Santis M., Bonfantini F., Di Salvo F., Dispinzieri M., Mantero E., Soncini F., Baili P., Sant M., Bianchi G., Maggi C. (2016). Factors influencing acute and late toxicity in the era of adjuvant hypofractionated breast radiotherapy. Breast.

[45] Moons K.G.M., Altman D.G., Reitsma J.B., Ioannidis J.P.A., Macaskill P., Steyerberg E.W., Vickers A.J., Ransohoff D.F., Collins G.S. (2015). Transparent Reporting of a multivariable prediction model for Individual Prognosis or Diagnosis (TRIPOD): Explanation and Elaboration. Ann. Intern. Med..

[46] Blanche P., Dartigues J.-F., Jacqmin-Gadda H. (2013). Review and comparison of ROC curve estimators for a time-dependent outcome with marker-dependent censoring. Biom. J..

[47] Trevethan R. (2017). Sensitivity, Specificity, and Predictive Values: Foundations, Pliabilities, and Pitfalls in Research and Practice. Front. Public Health.

